# Metabolic and endocrinologic interplay in the peri-ovulatory follicle to support the cumulus-oocyte-complex towards full competence

**DOI:** 10.1590/1984-3143-AR2025-0067

**Published:** 2025-08-18

**Authors:** Hilde Aardema, Peter L. A. M. Vos, Alberto Maria Luciano, José Buratini

**Affiliations:** 1 Farm Animal Health, Department of Population Health Sciences, Faculty of Veterinary Medicine, Utrecht University, Utrecht, The Netherlands; 2 Reproductive and Developmental Biology Laboratory, Department of Veterinary Medicine and Animal Sciences, University degli Studi di Milano, Milano, Italy; 3 In Vitro Equinos, Mogi Mirim, SP, Brasil; 4 Instituto de Biociências, Faculdade de Medicina Veterinária, Universidade Estadual Paulista “Júlio de Mesquita Filho” – UNESP, Botucatu, SP, Brasil

**Keywords:** free fatty acids, peri-ovulatory follicle, hormones, cumulus-oocyte-complex, final maturation

## Abstract

The increase in free fatty acid (FFA) levels in the circulation and follicular fluid in response to the negative energy balance of dairy cows has received significant attention during the last decades. However, until recently the potential effect of FFA on the periovulatory steroid environment has been overlooked. The well-orchestrated luteinizing hormone (LH) peak induces a steroid shift in the periovulatory follicle, from Estradiol-17β (E2) dominance around the LH peak towards progesterone (P4) dominance around ovulation, and is a prerequisite for optimal cytoplasmic and nuclear maturation in the oocyte and oocyte developmental competence. Recent insights in literature demonstrate a link between saturated and mono-unsaturated FFAs and the expression of gonadotrophin receptors, follicle stimulating hormone (FSH)R and LHR, including steroid related enzymes and E2 synthesis by in vitro granulosa cells. The current review will focus on the potential role of mono-unsaturated oleic acid, the most abundant FFA in follicular fluid, on steroidogenesis and its potential effect on the cumulus-oocyte-complex (COC) during final maturation. The data of this review suggest the potential for a regulatory interlinked system, which includes the oocyte secreted factor FGF10 and oleic acid, that modulates the steroidogenic switch from E2 to P4 in the periovulatory follicle, via actions that involve the extracellular signal-regulated kinase 1/2 (ERK1/2) pathway in support of the delicate and well-orchestrated dialogue between the oocyte and cumulus cells during final maturation of COCs.

## Introduction

Metabolic stress conditions, such as the negative energy balance (NEB) condition in cows, as well as obesity in humans, result in increased body fat mobilization and consequently elevated levels of free fatty acids (FFA), which are fatty acids complexed to albumin, in the blood circulation. The topic has received significant attention for the last two decades due to the existing relation between the metabolic impact of the NEB and reduced fertility performance in dairy cows (Britt, 1992; Butler and Smith, 1989; Diskin et al., 2006; Leroy et al., 2008; Sartori et al., 2002; Walters et al., 2002). Metabolic stress conditions like the NEB result in elevated levels of FFA in both blood and follicular fluid, dominated by three FFA: saturated stearic and palmitic acid, and mono-unsaturated oleic acid (Aardema et al., 2013a; Leroy et al., 2005). Some studies report a high level of linoleic acid, the most abundant poly-unsaturated FFA (~25-75 µM), but this is most probably due to the total fatty acid analysis that includes the high-density lipoproteins that are present in follicular fluid, and contain triacylglycerols (TG; with a glycerol backbone with three esterified fatty acids), and are rich in cholesterol-esters (CE; cholesterol with an esterified fatty acid) (Aardema et al., 2013a, 2018; Bender et al., 2010; Homa and Brown, 1992; Jungheim et al., 2011; Leroy et al., 2005; Wonnacott et al., 2010). A recent study in our group demonstrated that the CE fraction in the circulation of dairy cows, from peripartum until at least 16 weeks postpartum, indeed contains a high level of linoleic acid (Piscopo et al., 2025). In vitro studies on the effects of FFA have demonstrated a dose-dependent negative effect of in particular saturated FFA, like palmitic and stearic acid, via lipotoxic stress responses on somatic cells, including theca, granulosa, and cumulus cells (Cnop et al., 2001; Leroy et al., 2005; Listenberger et al., 2001, 2003; Maedler et al., 2001; Mu et al., 2001; Vanholder et al., 2005). The lipotoxic stress response by saturated FFA in somatic cells can be induced by ceramide formation, mitochondrial release of cytochrome-c, and caspase activation, resulting in reactive oxygen species (ROS) and increased apoptosis (Cnop et al., 2001; Coll et al., 2008; Henique et al., 2010; Listenberger et al., 2001, 2003; Maedler et al., 2001; Mishra and Simonson, 2005). Cumulus cells exposed to saturated FFA during COC maturation demonstrated increased ROS and apoptosis, via mechanisms such as ceramide formation, endoplasmic reticular stress, and impaired mitochondrial function (Leroy et al., 2005; Lolicato et al., 2015; Sutton-McDowall et al., 2016; Wu et al., 2012). The effects of saturated FFA on cells are in contrast to those observed for mono-unsaturated FFA, like oleic acid, which is in general harmless to cells and able to prevent lipotoxic events by saturated FFA, via the induction of lipid storage and fatty acid breakdown, via β-oxidation in mitochondria, in cells (Henique et al., 2010; Listenberger et al., 2003; Maedler et al., 2001). Also, in maturing COCs exposed to saturated FFA, mono-unsaturated oleic acid (C18:1) was shown to counteract the negative effects on oocyte developmental competence by the induction of lipid accumulation in cumulus cells (Aardema et al., 2013a, 2011; Lolicato et al., 2015). There appears to be a high level of tolerance by maturing COCs for mono-unsaturated oleic acid, the most abundant FFA in follicular fluid in dominant follicles (>14mm), with levels of up to 500 µM being harmless (Aardema et al., 2011, 2013a, 2015). However, this appears to be different for poly-unsaturated FFA. When maturing COCs were exposed to 50-100 µM of poly-unsaturated linoleic acid(C18:2), this relatively low concentration resulted in reduced ratios of metaphase II stage oocytes, cleavage, and blastocyst rates (Marei et al., 2010). The reduced percentage of metaphase II oocytes, resulting from a decrease in germinal vesicle breakdown, was consistent with a previous study in which COCs were exposed to linoleic acid (Homa and Brown, 1992). Poly-unsaturated linolenic acid (C18:3), at a concentration of 50 µM, improved cleavage and hence blastocyst rates compared to the control, whereas higher doses of linolenic acid, from 100 µM, had a detrimental effect (Marei et al., 2009; Marei et al., 2017). In the current review, we will focus on the potential link between mono-unsaturated oleic acid, given the high level of oleic acid in follicular fluid and high tolerance of COCs for this FFA, and the periovulatory steroid environment based on recent insights into the effects of FFA on steroidogenesis. During the follicular phase of final maturation, a ‘healthy peri-ovulatory follicular environment’, has been defined by an optimal ratio of steroid levels of Estradiol-17β (E2) and Progesterone (P4), and is a prerequisite for the growth and development towards a fully competent oocyte during the final phase of nuclear and cytoplasmic maturation (Aardema et al., 2013b; Dieleman et al., 1983; Kruip and Dieleman, 1985). The interplay between FFA in follicular fluid, and especially the abundant levels of oleic acid in the steroid environment of the follicle form the basis for the current review, as it might unravel an interesting potential link between the FFA composition and a healthy peri-ovulatory follicular steroid environment to support the delicate interaction between cumulus cells and the oocyte during the final maturation period.

## The steroid peri-ovulatory follicle environment is linked with oocyte competence

**Figure 1:**
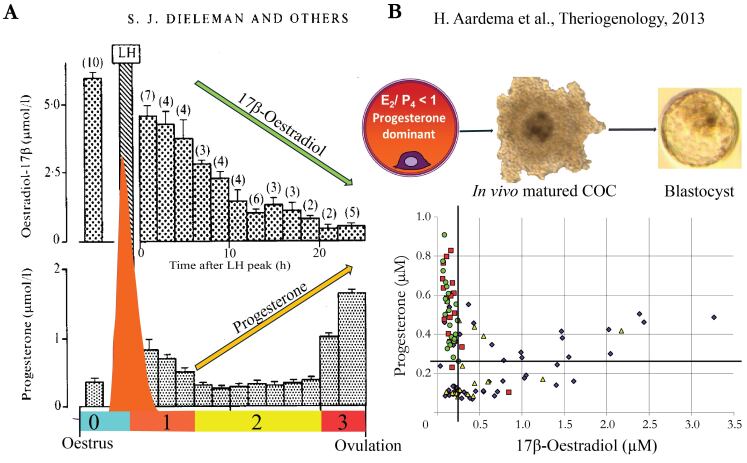
The switch in E2 towards P4 dominating levels is a prerequisite for oocyte developmental competence. The different phases (A) are defined on the basis of their steroid profile in time; Phase 0 the onset of oestrus until the LH surge, phase 1 the first 6h post LH, phase 2 from 6-20h post LH, phase 3 from 2h until ovulation (figure adapted from Dieleman et al., 1983); (B) represents the link at 22h post LH between E2 and P4 levels in periovulatory follicles and developmental competence of oocytes with blastocysts (green circles) exclusively from follicular fluid of low E2 (≤ 0.25 µM) and high P4 (≥ 0.26 µM), embryos ≥ 8 cells (red squares), embryos < 8 cells (yellow triangles) and non-cleaved (purple diamonds; figure adapted from Aardema et al., 2013b).

Former in vivo studies have demonstrated a link between a developmentally competent oocyte and a ‘healthy peri-ovulatory steroid environment’. The E2 produced from the precursor testosterone synthesized by theca and granulosa cells of the peri-ovulatory follicle, induces a GnRH surge and subsequently a follicle stimulating hormone (FSH) and luteinizing hormone (LH) surge, when progesterone levels are low, which sets the stage for an intriguing steroid shift at the level of the follicle prior to ovulation (Dieleman et al., 1983). The LH surge stimulates the differentiation of the theca and granulosa cells into luteal cells, which will switch the production from E2 to P4 dominance, and is accompanied by a decline in mRNA expression of the enzymes P450 17α-hydroxylase (CYP17; the rate-limiting enzyme for testosterone synthesis) and P450 aromatase (CYP19A1; the rate-limiting enzyme for E2 synthesis) (Chaffin et al., 2000; Fortune and Quirk, 1988; Komar et al., 2001; Murphy, 2000). The LH surge, occurring approximately 24h before the moment of oocyte ovulation, induces a massive drop in E2 level from 6h post LH surge, with concentrations of about 6 µM between the onset of oestrus and the LH peak (phase 0) decreasing to approximately 0.5 µM from 20h post LH until ovulation (phase 3). Meanwhile, there is a significant rise in P4 levels, increasing from approximately 0.4 µM (phase 0) to around 1.5 µM (phase 3), respectively (Dieleman et al., 1983). See Figure 1.

The decrease in E2 levels in follicular fluid corresponds to a significant ten-fold decrease in *CYP19A1* activity, when comparing granulosa cells collected at 10-15h post LH and those shortly before the LH peak, and a decrease in *CYP17.* Simultaneous with the luteinization of theca cells, resulting in the first P4 rise in response to the LH surge, with granulosa cells being responsible for the steady rise in P4 levels in follicular fluid towards the moment of ovulation (Dieleman et al., 1983; Fortune and Quirk, 1988; Komar et al., 2001). There are indications that E2 may play a role as a paracrine regulator, based on a study that demonstrated a decrease in P4 levels after in vitro E2 exposure of fetal bovine ovaries (Allen et al., 2016). Studies that have collected follicular fluid from follicles at different stages during the period of final maturation (ovaries collected by ovariectomy) in eCG superstimulated cows demonstrated a distinct link between steroid levels in follicular fluid and the synchronous cytoplasmic and nuclear maturation of oocytes, with matured metaphase II stage oocytes predominantly originating from P4 dominating follicles around ovulation (de Loos et al., 1991a; Dieleman et al., 2002; Hendriksen et al., 2000; Hyttel et al., 1986; Moorey et al., 2022). The E2 receptors ERβ and ERα are both expressed in cumulus cells, while only ERα is found in oocytes (Beker-van Woudenberg et al., 2004). Exposure to E2 during final oocyte maturation, however, resulted in a decreased rate of metaphase II (MII) oocytes, and a significantly higher number of nuclear abnormalities from 8h of maturation after germinal vesicle breakdown (GVBD), due to aberrant spindle formation compared to the control group (Beker-van Woudenberg et al., 2004). These data highlight the significance of reduced E2 levels during the final stage of oocyte maturation. In this respect, the steroid switch in peri-ovulatory follicles, towards a low level of E2 and a high level of P4 at the end of the final maturation phase, appears to be a prerequisite for the oocyte’s competence to reach the blastocyst stage (Aardema et al., 2013b). In the latter study, follicles were individually qualified based on E2 and P4 levels measured in the follicular fluid of preovulatory follicles in non-stimulated cows (Dieleman et al., 1983). After in vivo maturation the collected oocytes were individually in vitro fertilized and cultured to enable the retrospective linking of the follicle of origin to oocyte developmental competence (Aardema et al., 2013b). Hendriksen et al. stated already 25 years ago that ‘the history of the follicle determines the fate of the oocyte’ (Hendriksen et al., 2000). According to the steroid environment, this appears to be the case indeed. Interestingly, at 22h post LH, only those oocytes that originated from follicles with a low E2 (≤ 0.25 µM) and high P4 level (≥ 0.26 µM), related to a complete cumulus cell expansion, were able to develop into a blastocyst confirming the former hypothesized importance of E2/P4 <1 at the end of maturation (Aardema et al., 2013b). See also Figure 1.

## Potential link between FFA and steroid concentration in peri-ovulatory follicles

The first indication of a potential link between FFAs and steroids in the peri-ovulatory follicle originates from different FFA compositions in deviant and healthy follicles, as determined by the levels of E2 and P4 in follicular fluid, in our former dataset. Remarkably, follicular fluid derived of presumptive ovulatory follicles qualified as deviant at the end of maturation (E2/P4 >1) contained relatively high levels of stearic acid and low levels of oleic acid (respectively, 17.09 ± 1.65% and 28.34 ± 3.72%; Aardema et al., unpublished data), in contrast to healthy follicles with low levels of stearic (8.68 ± 0.96%) and high levels of oleic (44.05 ± 1.99%; (Aardema et al., 2013a). This is of interest as Stearoyl-CoA desaturase (SCD), the enzyme that converts saturated stearic acid into mono-unsaturated oleic acid, is highly expressed in granulosa and cumulus cells. A higher SCD expression and activity results in higher levels of oleic acid, as observed in healthy follicles, and may relate to the steroid switch from E2 and P4 dominance during final maturation (see later). Superstimulated heifers that experienced metabolic stress, resulting in increased levels of FFA, palmitic, stearic and in particular oleic acid (~ 2x higher) in follicular fluid, had a significantly lower number of large follicles (> 12 mm; 2.7 ± 0.9) at 22 h after the induced LH surge, versus those in the control group (6.0 ± 1.7) combined with lower E2 levels (Aardema et al., 2010, 2013a, 2019). In line with a previous study in our group with synchronized, not superstimulated, metabolically stressed heifers having smaller sized follicles with decreased E2 levels in follicular fluid, and lower E2/P ratio in comparison to control cows (Jorritsma et al., 2003). In combination with the doubling of oleic acid in the follicular fluid of metabolically stressed animals, this might suggest a potential negative link between oleic acid and E2 levels in the follicular fluid. Several studies in cows have indeed reported that the fatty acid profile of follicles appears to be related to E2 levels in follicular fluid. An early bovine study of Moallem et al. reported significantly higher levels of oleic and linoleic acid, and lower levels of palmitic acid in dominant follicles (> 8 mm) that were defined as inactive or less active, based on low E2 levels, after cows were treated with calcium soaps of fatty acid or bST (Moallem et al., 1999). Also in a study of Renaville et al. where postmortem follicles (> 8 mm) were collected from cows in dioestrus, defined as active (E2/P4 > 1) or inactive (E2/P4 < 1), the FFA profile in follicular fluid was different between active and inactive follicles with a relatively higher level of palmitic acid and oleic, and lower level of stearic acid in active follicles (Renaville et al., 2010). In a study where dominant follicles (Ø 7-8 mm) were injected with a FFA mixture of palmitic, stearic and oleic acid (each at 200 µM), via transvaginal ultrasound guidance in synchronized cows, this resulted in lower follicle diameters at 24 and 48 h post injection, versus the control group that received a vehicle. Still, the FFA mixture had no significant effect on E2 levels or the expression of steroidogenic-related genes in granulosa cells (Ferst et al., 2020). However, when oleic acid was injected in dominant follicles (Ø 10-19 mm) this resulted in a significant drop in E2 levels, compared to the control group that received a BSA vehicle, and a reduced expression of *CYP19A1* and *steroidogenic acute regulatory* (*STAR*; Sharma et al., 2019). With STAR being the enzyme that regulates the rate limiting step of steroidogenesis by transporting cholesterol from the outer to inner mitochondrial membrane. Periparturient supplementation of fats with low or high levels of, rumen bypass, unsaturated fatty acids (oleic and linoleic), in contrast, resulted in larger preovulatory follicles between day 50 and 75 postpartum, compared to the group fed with low levels of unsaturated fatty acids, and higher levels of E2 and increased *CYP19A1* expression in granulosa cells (Zachut et al., 2008). Furthermore, when lactating dairy cows were fed with different sources of fatty acids, the number of oocytes collected from the group that received sunflower oil, rich in oleic acid (80%), was significantly higher compared with those fed with trans oleic acid, linoleic acid, or linolenic acid (Bilby et al., 2006). This is in line with our recent periparturient fat supplementation study that resulted in a significantly higher number of follicles and consequently higher oocyte yield (1.6 times) collected at 8, 12 and 16 weeks postpartum for cows fed with an oleic acid rich fat-supplementation versus the control group that received a standard fat supplement rich in palmitic acid (Piscopo et al., 2025). The above data suggest that oleic acid appears to be related to E2 levels in the follicle, but that the effect on follicles during final maturation might differ in comparison to earlier stage follicles.

## Steroid regulation in follicular granulosa cells is differently affected by saturated and unsaturated FFAs

**Figure 2:**
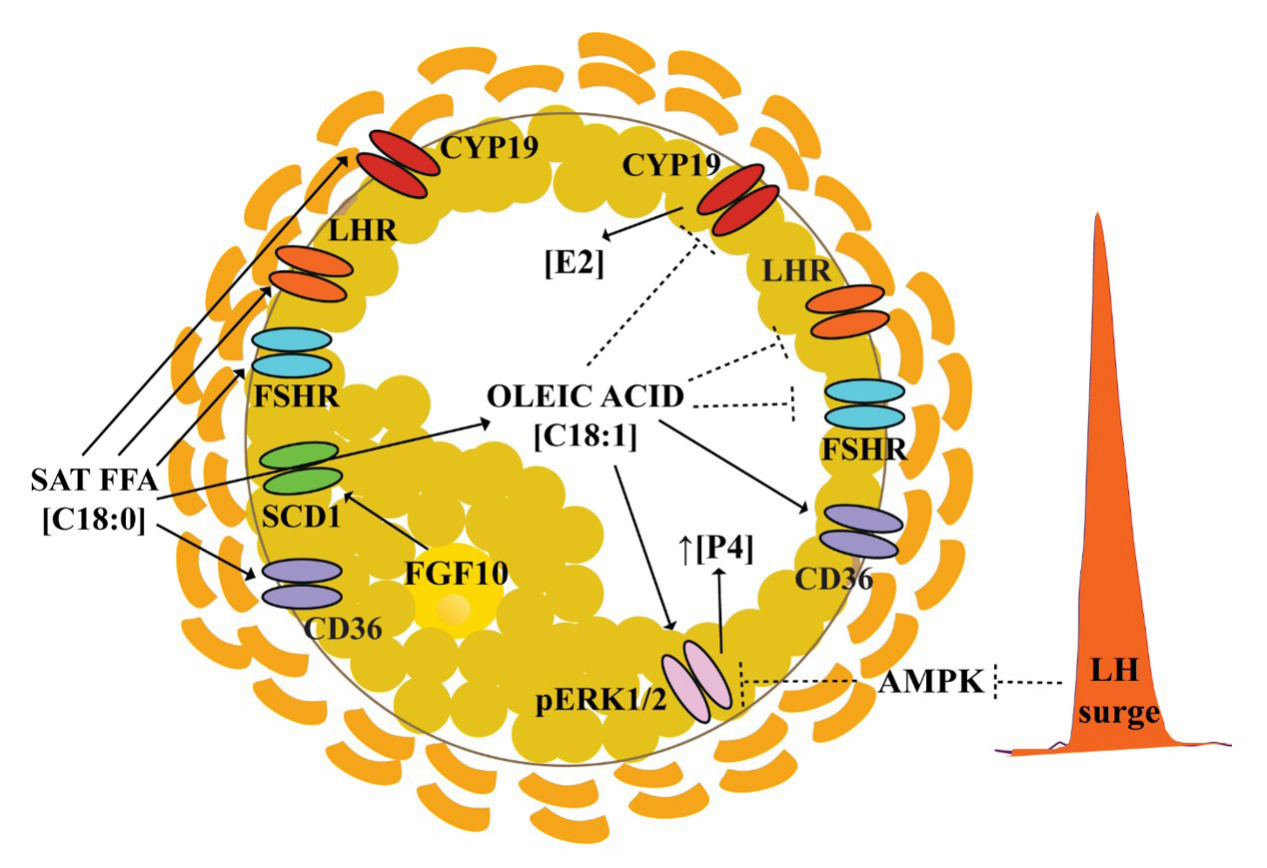
Schematic picture representing the most significant effects of oleic acid and saturated FFA after in vitro granulosa cell exposure on steroidogenesis. Saturated free fatty acids (FFA), including stearic acid (C18:0) increase the expression of gonadotrophin receptors, FSHR and LHR, and aromatase (CYP19) resulting in increased Estradiol-17β (E2) synthesis. In contrast, oleic acid induces a downregulation of FSHR and LHR, and CYP19, which results in a drop of E2 synthesis. Furthermore, oleic acid significantly increases pERK1/2 that appears to be required for an increased synthesis of progesterone (P4) by granulosa cells. When the AMPK block of pERK1/2 is released under the influence of the LH surge, we speculate that pERK1/2 can be activated by the FGF10 – SCD1 – Oleic acid pathway.

Despite the well-known complementary and required steroidogenic step in theca cells of the follicle from cholesterol towards testosterone (androstenedione), which precedes the production of E2 by granulosa cells, this review will primarily focus on the response of granulosa cells to FFA in relation to steroidogenesis and the potential effect on the steroid environment of the COC, because: 1) most available literature in relation to FFA focuses on granulosa cells and 2) granulosa cells precede the more differentiated state of the specialized cumulus cells connected to the oocyte. In vitro studies investigating the effect of unsaturated oleic acid exposure on granulosa cells mostly report reduced proliferation and diminished expression of *cyclin-D2* (*CCND2*), and changes in the morphology, whereas saturated FFA appear to have no adverse effect on cell proliferation (Baddela et al., 2022a; Yenuganti et al., 2016, 2021; Zhou et al., 2022). However, consistent with findings for other somatic cell types (see above), a number of studies demonstrate an increased level of apoptosis for saturated FFA in granulosa cells (Mu et al., 2001; Vanholder et al., 2005), whereas one study also reports a mild increase of apoptosis in response to oleic acid (<10%; (Baddela et al., 2022b). Interestingly, gene analysis revealed morphological and structural changes in granulosa cells after oleic acid exposure, suggesting a cellular switch from a follicular to luteal transition (Yenuganti et al., 2021). These data indicate a potential role for oleic acid in the LH surge-induced switch from follicular to luteal P4-producing cells in the periovulatory follicle and certainly deserve attention in future studies. Interestingly, in vitro exposure of granulosa cells, collected from mid-antral follicles (Ø 2-6 mm), to mono-unsaturated oleic acid resulted in a significant dose-dependent downregulation of E2 synthesis by granulosa cells, in contrast to exposure to saturated palmitic and stearic acid, which resulted in increased levels of E2 (Baddela et al., 2022a; Sharma et al., 2019; Yenuganti et al., 2016, 2021; Zhou et al., 2022). A study with granulosa cells collected from dominant follicles (Ø >8 mm), in contrast, reported an increase in E2 levels after exposure to a high, non-physiological, oleic acid concentration of 500 µM, and not for 150, 300 µM (Vanholder et al., 2005). The consistently reported downregulation in E2 synthesis in granulosa cells after exposure to oleic acid is linked to a dose-dependent downregulation of the expression of gonadotrophin receptor *FSHR*, and steroidogenic-related genes, *STAR*, *3β-Hydroxysteroid dehydrogenase* (*HSD3B)*, *Cholesterol side-chain cleavage enzyme* (*CYP11A1)*, *CYP19A1,* and also a downregulation of *LHR* (≥ 200 µM; (Baddela et al., 2022a; Sharma et al., 2019; Yenuganti et al., 2016, 2021). See Table 1 and Figure 2.

**Table 1:** Overview of main effects of in vitro granulosa cells in response to FFA.

**Granulosa cell**		**+ Oleic acid**		**+ Stearic or Palmitic**		**+ Mix FFA**
**Response**		**(mono-unsaturated)**		**(saturated FFA)**		**(Oleic acid & saturated)**
**E2 synthesis**	**↓↓**	Yenuganti et al. (2016) Sharma et al. (2019) Yenuganti et al. (2021) Baddela et al. (2022a) Zhou et al. (2022) (porcine)	**↑**	Sharma et al. (2019) (From 200µM) Baddela et al. (2022a) (only at 50µM)	**↓**	Sharma et al. (2019)
	**=**	Vanholder et al. (2005)	**↑**	Vanholder et al. (2005)	**↑**	Vanholder et al. (2005)
					=	Baddela et al. (2022a)

**P4 synthesis**	**↑**	Coyral-Castel et al. (2010) (goat)					


	**=/↓**	Yenuganti et al. (2016) and Sharma et al. (2019) (↓ Only 400µM) Baddela et al. (2022b) (**↓** at 200 and 400µM) Zhou et al. (2022) (porcine) (**↓** at 300 and 500µM)	=	Sharma et al. (2019) Baddela et al. (2022b)	=	Sharma et al. (2019)	
***FSHR***	**↓↓**	Yenuganti et al. (2016) Sharma et al. (2019) Yenuganti et al. (2021) Baddela et al. (2022a)	**↑**	Sharma et al. (2019) Baddela et al. (2022a)	=	Sharma et al. (2019) Baddela et al. (2022a)	
***LHR***	**↓**	Yenuganti et al. (2016) Sharma et al. (2019) Yenuganti et al. (2021)	**↑**	Sharma et al. (2019) Baddela et al. (2022a)	**↑**	Sharma et al. (2019)	
					**=**	Baddela et al. (2022a)	
***CYP11A1***	**↓**	Yenuganti et al. (2016) Baddela et al. (2022b) Zhou et al. (2022) gene and protein (porcine)			=	Baddela et al. (2022b)	
***CYP19A1***	**↓↓**	Yenuganti et al. (2016) Sharma et al. (2019) Yenuganti et al. (2021) Zhou et al. (2022) gene and protein (porcine)	**↑**	Sharma et al. (2019)	=	Sharma et al. (2019) Baddela et al. (2022a)	
			**=**	Baddela et al. (2022a)	↓	Zhao et al. (2025) (gene and protein)	
***HSD3B1***	**↓**	Yenuganti et al. (2016) and Sharma et al. (2019) (only 400 µM) Baddela et al. (2022b)	**↑**	Sharma et al. (2019) ≥ 100 µM palmitic, and ≥ 200 µM stearic acid	=	Sharma et al. (2019) Baddela et al. (2022b)	
					**↓**	Zhao et al. (2025) (gene and protein)	
**STAR**	**↓**	Yenuganti et al. (2016) and Sharma et al. (2019) (At 400 µM oleic) Zhou et al. (2022) (gene and protein, porcine)	**↓**	Sharma et al. (2019)	**↓**	Sharma et al. (2019) Zhao et al. (2025) (gene and protein)	

**CD36**	**↑↑**	Yenuganti et al. (2016) and Sharma et al. (2019) Yenuganti et al. (2021) Baddela et al. (2022a) Zhou et al. (2022) (porcine)	**↑**	Sharma et al. (2019) (≥ 200µM) Baddela et al. (2022b)	**↑**	Sharma et al. (2019)	
					**=**	Baddela et al. (2022b)	
**pERK**	**↑↑**	Yenuganti et al. (2021) Baddela et al. (2022a) Tao et al. (2024) Tosca et al. (2005) (rat) Coyral-Castel et al. (2010) (goat)	=	Baddela et al. (2022a) Tao et al. (2024)	=	Baddela et al. (2022a) Tao et al. (2024)	
**pAKT**	**↑**	Baddela et al. (2022a) Tao et al. (2024)	=	Baddela et al. (2022a) Tao et al. (2024)	=	Baddela et al. (2022a) Tao et al. (2024)	
**CCND2**	**↓**	**Yenuganti et al. (2016)** Sharma et al. (2019) **Zhao et al. (2025)**					
**Apoptosis**	**=**	Mu et al. (2001) (human) Sharma et al. (2019) Baddela et al. (2022a)	**↑**	Mu et al. (2001) (human) Vanholder et al. (2005)	**↑**	Zhao et al. (2025)	
	**↑**	Baddela et al. (2022b) (< 10% apoptosis)	**=**	Sharma et al. (2019) Baddela et al. (2022a) Baddela et al. (2022b)			
**Morphology**	**+**	Yenuganti et al. (2016) and Sharma et al. (2019) (At 400 µM oleic) Yenuganti et al. (2021) Baddela et al. (2022b)	**+**	Sharma et al. (2019) (At 400 µM oleic) Mu et al. (200 1);	+	Sharma et al. (2019)	

The table summarizes data that are predominantly based on studies on bovine granulosa cells, it is, between brackets, notified when non-bovine species are studied or effects are only present at a certain concentration. Arrows ↓ refer to a significant downregulation, and ↑ to an upregulation. No significant effect, in comparison to the control group in the reported study, is visualized with =. Estradiol- 17β (E2), Progesterone (P4), Follicle stimulating hormone receptor (FSHR), Luteinizing hormone receptor (LHR), Cholesterol side-chain cleavage enzyme (CYP11A1), P450 aromatase (CYP19A1), 3β-Hydroxysteroid dehydrogenase (HSD3B), steroidogenic acute regulatory (STAR), long chain free fatty acid transporter (CD36), phosphorylated extracellular signal-regulated kinase (pERK), and phosphorylated protein kinase B (pAKT).

## The effects of unsaturated FFAs on steroidogenesis are counteracted by saturated FFAs

Exposure of granulosa cells to saturated palmitic and stearic acid resulted in a dose-dependent upregulation of *LHR* and *FSHR*, and an upregulation of *CYP19A1* and *HSD3B,* with saturated FFAs being able to counteract the by oleic acid induced downregulation on gonadotrophin receptors and steroidogenic genes and the restored levels of E2 (Baddela et al., 2022a; Sharma et al., 2019). A study performed with a FFA mixture consisting of five different FFA, oleic (~ 43%), palmitic (32%), stearic (15%), linoleic (5%) and palmitoleic (5%) at a total concentration of 400 µM, reported a downregulation for both *gene* and protein expression of *CYP19A1, STAR and HSD3B,* with the adverse effects of the FFA mixture on granulosa cells being tempered by the activation of *pAMPKɑ* (Zhao et al., 2025). Furthermore, the study of Zhao et al. reported downregulation, which seems in contrast to another study in granulosa cells where a mixture with unsaturated oleic (100 µM), linoleic (100 µM), and ɑ-linolenic (100 µM) acids resulted in reduced E2 levels, but not in combination with saturated palmitic (100 µM) and stearic acid (100 µM) (Baddela et al., 2022a). A study with a mixture of high levels of unsaturated oleic (400 µM), combined with saturated palmitic (200 µM) and stearic acid (200 µM), did, however, despite the presence of saturated FFA in the mixture, report a decrease in E2 levels and *STAR* expression (Sharma et al., 2019). The distinct levels of FFA and different ratios of unsaturated and saturated FFA may largely determine the final effect on steroidogenesis in the follicle. Despite the apparent link between FFA exposures and the steroidogenic response in granulosa cells, we should nevertheless be careful with the interpretation of the *in vitro* generated data in studies as 1) the exposure levels generally used, e.g. oleic acid exposures, are relatively high in relation to the physiological ranges and 2) most do not resemble the FFA mixture that is normally present in follicular fluid and 3) most are performed with granulosa cells collected of medium sized antral follicles (Ø<6 mm), and the response of granulosa cells to FFA might strongly depend on the presence of FSHR. The only study we are aware of that used granulosa cells of dominant follicles (>8 mm) for in vitro culture reported a dose-dependent increase in E2 in response to oleic acid (Vanholder et al., 2005). Exposure to low doses of oleic acid resulted in an increase in P4 synthesis by granulosa cells (Coyral-Castel et al., 2010). In contrast, others showed that oleic acid (up until 200 µM) with or without saturated FFAs had no effect on the P4 levels but, higher doses and a non-physiological dose of 400 µM oleic acid resulted in a drop in P4 levels (Sharma et al., 2019; Yenuganti et al., 2016). An earlier study demonstrated that P4 synthesis in rat granulosa cells depends on extracellular signal-regulated kinase (*pERK1/2)* activation, with P4 synthesis being inhibited by *AICAR*, which activates *AMPK*. This inhibition most likely occurs through the downregulation via 3β-HSD protein and gene, with no effect on *CYP19A1* and E2 synthesis (Tosca et al., 2005). The role of AMPK in blocking steroidogenesis upon activation by LH has also been demonstrated in another study, where LH-induced P4 synthesis was prevented in the presence of AMPK (Przygrodzka et al., 2021). At the same time, the expression of *3β-HSD*, *STAR*, and *CYP11* was not affected by AMPK (Przygrodzka et al., 2021). See Table 1 and Figure 2.

## Unsaturated oleic acid activates the pERK1/2 pathway in granulosa cells

Interestingly, in vitro oleic acid exposure resulted in granulosa cells, independent of the concentration (≥ 10 µM) and within one minute of stimulation, in a significant increase in the expression of *pERK 1/2*, whereas there was no effect on *pERK 1/2* after exposure to saturated FFA or a FFA mixture (Baddela et al., 2022a; Coyral-Castel et al., 2010; Tao et al., 2024; Yenuganti et al., 2021). In goat granulosa cells, it has been demonstrated that p*ERK 1/2* activity is a prerequisite for oleic acid-induced P4 synthesis (Coyral-Castel et al., 2010). Also, in bovine granulosa cells, increased *pERK1/2* appears to be linked to steroidogenesis, characterized by *FOXL2* downregulation and *SOX9* upregulation, resulting in increased *STAR* protein expression and P4 synthesis as well as a downregulation of *CYP19A1* and E2 synthesis compared to the group where *ERK1/2* was inhibited (Baddela et al., 2023). The by oleic acid stimulated *pERK* pathway in granulosa cells has also been reported for other somatic cell types, including bovine mammary epithelial cells, breast cancer cells, and smooth muscle cells where exposure to oleic acid likewise resulted in a rapid increase in *pERK* (Matoba et al., 2018; Soto-Guzman et al., 2008; Yonezawa et al., 2008). The by oleic acid induced downregulation of gonadotrophin receptors, *FSHR* and *LHR*, and activation of the *pERK* pathway resulting in reduced E2 synthesis and increased P4 synthesis seems to indicate that oleic acid has an important modulatory role in the activation of the switch from E2 towards P4 dominance in the periovulatory follicle, after the AMPK block on the pERK pathway is gone. See Table 1 and Figure 2.

## Stearoyl-CoA desaturase activity appears to be linked to E2 synthesis

Stearoyl-CoA desaturase (SCD), also known as Δ9 desaturase, is the key regulatory enzyme that controls the ratio of mono-unsaturated and saturated FFA in the body. SCD is localized at the membrane of the endoplasmic reticulum and catalyzes the rate-limiting step towards the formation of mono-unsaturated fatty acids, such as oleic acid, from, e.g., saturated stearic acid. This process involves the introduction of a cis-carbon–carbon double bond at the Δ 9 position of the hydrogen carbon chain. There are distinct SCD types that differ among species, despite their similar desaturase function. In Muridae four different genes code for SCDs: *SCD1, SCD2, SCD3, and SCD4* (Flowers and Ntambi, 2008; Maedler et al., 2001), and in e.g. human and bovine two SCD genes: SCD1 and SCD5 (Ntambi et al., 1988; Wang et al., 2005). *SCD* is abundantly expressed in somatic cells in the body, e.g. liver and mammary gland, and is highly expressed in the ovary in both granulosa and cumulus cells, with a weaker expression in theca and no detectable expression in the oocyte (Aardema et al., 2017; Fayezi et al., 2018; Feuerstein et al., 2007; Moreau et al., 2006; Pawlak et al., 2020; Warzych et al., 2017a, b). Since SCD converts saturated stearic acid (C18:0) into unsaturated oleic acid (C18:1), SCD activity in the follicle might be a primary driver for the demonstrated distinct FFA profile between blood and follicular fluid with relatively high levels of oleic acid and low levels of stearic acid (Aardema et al., 2013a, 2015; Leroy et al., 2005), rather than the by some suggested selective uptake of FFA by the follicle. The expression of *SCD1* is induced by, e.g., high-carbohydrate diets, whereas polyunsaturated FFA like linoleic acid inhibit *SCD* expression. Transcriptional control of SCD is mediated by transcription factors, including *liver X receptor* (*LXR*) and *SREBP1c*, as well as E2 (Liu et al., 2011). SCD expression in rat granulosa cells appears to be induced by FSH (Moreau et al., 2006). SCD1 has been recognized as a key factor in cancer studies, and its inhibition has been proposed as a potential cancer treatment, due to the reported link between cell proliferation and SCD activity (Ide et al., 2013; Scaglia and Igal, 2008). In an E2 receptor-positive breast cancer study, it was found that E2 stimulated SCD1 activity, as evidenced by the fact that SCD inhibition blocked cell proliferation (Belkaid et al., 2015). In a study on non-small cell lung cancer, where SCD1 activity promoted tumor metastasis via increased *CYP19A1* expression and E2 synthesis (Chen et al., 2023). These data from cancer studies suggest a direct link between SCD activity and E2 synthesis.

## Stearoyl-CoA desaturase activity in cumulus cells protects the oocyte

Former studies in our group demonstrated the importance of SCD1 activity in cumulus cells of COCs to be able to protect the oocyte against the toxic effects of saturated FFAs, as demonstrated by SCD1 inhibition of COCs that resulted in a significant drop in blastocyst rates in the presence of stearic acid (Aardema et al., 2017). In human cumulus cells, where both *SCD1* and *SCD5* genes are expressed, SCD1 has been suggested to be a potential predictive marker for oocyte competence (Fayezi et al., 2018; Feuerstein et al., 2007). Interestingly, and in line with the above data from cancer studies, a similar link is also observed in cumulus cells where SCD activity, and consequently increased levels of oleic acid, are associated with E2 synthesis. When isolated cumulus cells were exposed to SCD inhibition 48h before co-culture with denuded oocytes, this resulted in a negative impact on the expression of *CYP19A1* and E2 production in cumulus cells, leading to significantly lower rates of MII oocytes (Fayezi et al., 2018). The downregulation of *CYP19A1* and E2 synthesis by SCD inhibition in cumulus cells, which impaired oocyte developmental competence was entirely restored by the addition of 50 µM oleic acid (Fayezi et al., 2018). These data suggest considering SCD and oleic acid, the product of SCD activity, together as potential modulators of steroidogenesis at the level of the COC. The significant rise in SCD expression in cumulus cells from the germinal vesicle stage to MII stage oocytes in both human and bovine COCs, following the same expression pattern as *AREG*, is of interest (Aardema et al., 2017; Feuerstein et al., 2007). In particular, oleic acid, the end product of SCD activity, activates *pERK1/2* in granulosa cells (Coyral-Castel et al., 2010; Yenuganti et al., 2021; Baddela et al., 2022a; Tao et al., 2024). The previously reported *pERK1/2* upregulation in granulosa cells in response to oleic acid appears to be in line with preliminary data obtained in our group. Exposure to saturated FFA did not increase the levels of EREG in maturing COCs, but the combination with oleic acid resulted in significantly increased levels of *EREG* (Piscopo et al., unpublished data), with EREG being one of the ligands activating the *pERK1/2* pathway. Oleic acid, potentially via SCD activation, in the follicular fluid might be an important modulator for the required activation via pERK1/2 to make the switch from E2 dominance towards P4 dominance, and to set the stage for the final maturation steps that need to occur for the COC, when AMPK levels drop after the LH surge and the former AMPK block on ERK1/2 activation is released. See Figure 2.

## The FGF10/SCD/Oleic acid pathway in support of final maturation events in the follicle

Another factor to consider is fibroblast growth factor 10 (FGF10). FGF10 appears to induce SCD activity, as previously reported in embryonic epithelial lung cells (Lü et al., 2005). Interestingly, FGF10 is a paracrine factor expressed by both the oocyte and theca cells that inhibits E2 synthesis in granulosa cells, which express FGF10 receptors (FGFR1B and FGFR2B), very likely through the suppression of *FSHR* and *CYP19A1* expression (Buratini et al., 2007; Castilho et al., 2017). Furthermore, high levels of E2 in the follicular fluid coincided with decreased expression of *FGF10* (Buratini et al., 2007). Therefore, expression patterns and functional data indicate a role for the FGF10/SCD/Oleic acid axis during the switch from the E2 towards the P4 steroidogenic mode in the periovulatory follicle. On the other hand, given the ability of oleic acid to stimulate *pERK,* most likely via AREG/EREG signaling, the FGF10/SCD/oleic acid pathway may also play a role in the regulation of final COC maturation. See Figure 2.

## Cumulus cells are exposed to FFA in follicular fluid and form a shield around the oocyte

So far, most studies on FFA have focused on the potential adverse effects of metabolic stress during the final maturation of the COC. Oocytes originating from COCs that are exposed to saturated, stearic and palmitic acid during in vitro maturation are hampered in their developmental competence, whereas oleic acid exposure was harmless even at high levels (Aardema et al., 2011; Leroy et al., 2005; Lolicato et al., 2015; Sutton-McDowall et al., 2016; Wu et al., 2012). From the dominant follicle stage (Ø ≥ 14mm), there is no information yet on the FFA profile in smaller antral follicles, COCs are surrounded by oleic acid rich follicular fluid, independent of the presence of a NEB or the timing during the lactation period, with cumulus cells that form a barrier between the follicular fluid and oocyte (Leroy et al., 2005; Aardema et al., 2013a, 2015). Previous studies in our group reported a massive accumulation of lipids in cumulus cells while oocytes remained unaffected in terms of developmental competence (Aardema et al., 2013a). Fatty acids are an important source that can be efficiently stored in lipid droplets as TG and are; broken down via β-oxidation in mitochondria for energy, important building blocks for membranes in phospholipids, and transcription factors (Walther and Farese, 2012). In oocytes, the lipid content varies depending on the species, ranging from low in mice and humans to high in cattle, horses, and pigs. This variation has been linked to a longer duration until embryo implantation in species with high lipid content (McKeegan and Sturmey, 2011). Interestingly, lipid composition in oocytes itself has been linked to developmental competence; with the most abundant fatty acid in both A and B quality ranked oocytes being palmitic acid, but the second fatty acid in A ranked oocytes being oleic acid whereas it was stearic in B ranked oocytes (Kim et al., 2001). In the oocyte, lipid droplets and mitochondria are clustered together in so-called metabolic units, and the necessity of β-oxidation and a reduction of TG stores indicate that fatty acids are an important energy source during early development (Abe et al., 1999; Downs et al., 2009; Dunning et al., 2010, 2011; Ferguson and Leese, 1999, 2006; Hyttel et al., 1997; Kruip et al., 1983; McEvoy et al., 2000; Sturmey et al., 2006). Fatty acids can enter the oocyte, demonstrated by exposure of in vitro maturing COCs to RA labeled palmitic or oleic acid, which was followed by the incorporation of TG in lipid droplets and phospholipids of membranes from the oocyte and indicates active metabolization of fatty acids, with a preference for the uptake of oleic acid, ~30 ± 5% versus palmitic acid (Aardema et al., 2011). Cumulus cells appear to buffer and regulate the transfer of fatty acids towards the oocyte (Aardema et al., 2013a; Del Collado et al., 2017; Lolicato et al., 2015). The transzonal projections (TZPs) that connect cumulus cells and oocytes contain fatty acid binding proteins (FABPs) to facilitate the transport of long-chain FFAs like palmitic and oleic acid from cumulus cells towards the oocyte, with the disruption of the TZPs acting on actin filaments through cytochalasin resulting in a significant drop in the lipid amount of oocytes (Del Collado et al., 2017). This indicates active transfer of fatty acids from cumulus cells via TZPs to the oocyte. Furthermore, the presence of FABP significantly increased during the first 9h of maturation but remained stable in the following period from 9 until 18h of maturation together with stable lipid droplet numbers, after an initial increase in lipid droplet numbers during the first 9h of maturation (Del Collado et al., 2017). A previous study by Macaulay demonstrated that TZPs retract at 9 hours after the resumption of meiosis, which would indeed prevent the transfer of fatty acids from cumulus cells to the oocyte via the TZPs (Macaulay et al., 2014). These observations indicate a pivotal role of cumulus cells and collaboration with the oocyte in regulating fatty acid transfer via TZPs, including the option to form a more intense barrier of cumulus cells between the oocyte and follicular fluid once the TZPs are retracted, which might be a potential option to ‘isolate’ the oocyte from a rapidly changing periovulatory environment after the LH surge.

## Oleic acid might favor the modulation of the ERK pathway in COCs at final maturation

Cumulus cells support the oocyte towards full developmental competence (Albertini et al., 2001; Buratini et al., 2022; Gilchrist et al., 2008; Luciano et al., 2011), and are metabolically connected to the oocyte via these TZPs and functionally open gap-junction-mediated communication (Albertini et al., 2001; de Loos et al., 1991b; Del Collado et al., 2017; Li and Albertini, 2013). However, more recently, it has become clear that the oocyte primarily orchestrates the communication and transfer between the oocyte and cumulus cells. The TZPs that connect cumulus cells and oocytes are not only used for the exchange of nutrients like pyruvate, for which the oocyte depends on cumulus cells, but also for RNA transfer and communication factors, such as the oocyte secreted factors (OSF) of BMP15 and GDF9 (Biggers et al., 1967; Caixeta et al., 2013; Sugiura et al., 2005). Open gap-junction-mediated communication appears to be a prerequisite to maintain transcriptional activity for building up RNA stores, as long as possible, for protein synthesis to overcome the period until embryos gain transcriptional activity, with gradual chromatin condensation in germinal vesicle stage oocytes to mature towards a fully competent oocyte (Garcia Barros et al., 2023; Lodde et al., 2008; Luciano et al., 2011). Interestingly, studies investigating the role of the oocyte in relation to cumulus cell function demonstrate that cumulus cells lacking oocyte guidance behave differently. The delicate communication between cumulus cells and the oocyte depends on the presence of TZPs, which facilitates the establishment of gap-junction-mediated communication (Clarke, 2022). Their functionality is supported by gonadotropins and, more specifically, low levels of FSH that evoke low concentrations of cAMP (Luciano et al., 2004; Modina et al., 2001) and promote the maintenance of transcriptional activity and the achievement of developmental competence (Franciosi et al., 2014; Luciano et al., 2011) along with the role of high levels of AMH during the final stages of oocyte maturation. On the contrary, high levels of FSH decrease the gap junctional communication between oocyte and cumulus cells and reduce the TZP density, thus diminishing the communication between somatic and germinal compartments (Buratini et al., 2022, 2023; Combelles et al., 2004; Luciano et al., 2011). To maintain meiotic arrest in oocytes until full competence has been reached, the cGMP produced by cumulus cells under NPPC stimulation (Franciosi et al., 2014) and transferred to the oocyte is essential, as cGMP prevents the degradation of cAMP by phosphodiesterase in the oocyte (Conti et al., 2012; Zhang et al., 2010). TZP retraction has been suggested to occur in response to the LH surge, mediated by epidermal growth factor (EGF) receptor signaling (Abbassi et al., 2021). Based on data that was, for over 20 years, long neglected by Combelles et al. (2004), it has recently been hypothesized that excessive FSH signaling is a key mechanism underlying fertility decline in advanced maternal age. Intracellular pathways linking FSHR activation with TZP detachment from the oolemma and retraction, particularly EGFR transactivation leading to increased ERK1/2 activity (Richards, 1994; Sirard, 2019), have been identified, providing further support for this hypothesis. Interestingly, in parallel, the proponents of this hypothesis have demonstrated that oocyte secreted factors (OSFs) inhibit the abundance of mRNA encoding the *FSHR, AREG,* and *EREG* genes, while enhancing transcription of *NPPC* and *AMH* in cumulus cells (Buratini et al., 2023; Lima et al., 2016), suggesting efforts from the oocyte to reduce the pace of its own maturation and to preserve its communication with cumulus cells.

**Figure 3:**
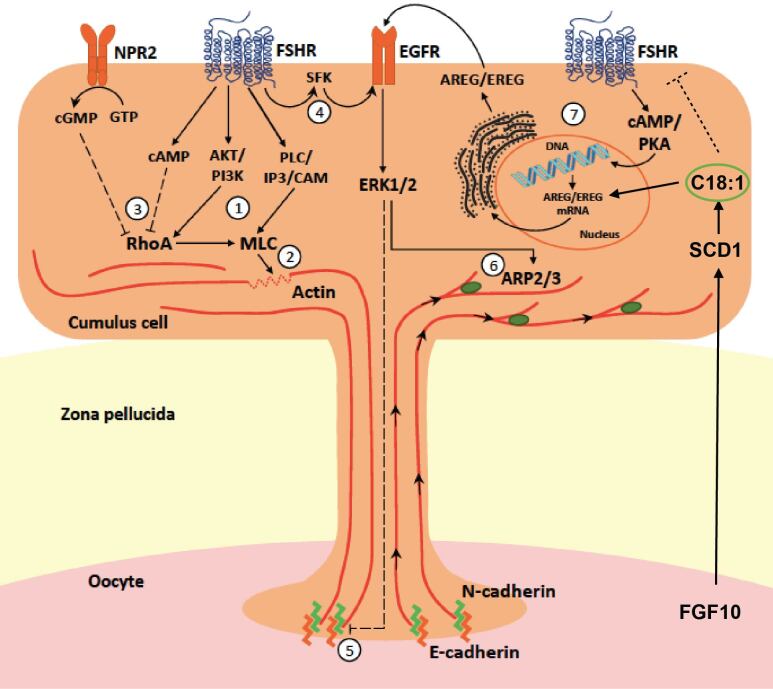
Hypothetical model of the FGF10/SCD/Oleic acid induced actions at the level of the cumulus-oocyte-complex and the effect on TZP dynamics. FGF10 induces SCD activation and the formation of oleic acid, which results in a downregulation of FSHR and activates the pERK1/2 pathway via increased expression of AREG/EREG. FSH signaling causes MLC phosphorylation more directly through either PLC/IP3/CAM or via RhoA activation through AKT/PI3K [1], with both pathways promoting actin reorganization [2]. Increased cAMP and cGMP intracellular levels resulting from FSHR and NPR2 (NPPC receptor) activation, respectively, inhibit RhoA/MLC, promoting actin stabilization [3]. FSH transactivates EGFR via SRC tyrosine kinases (SFK) [4], leading to ERK1/2 phosphorylation that promotes TZP detachment through N-cadherin/E-cadherin disruption [5], and actin contraction through ARP2/3-induced filament branching [6]. FSH indirectly promotes EGFR canonical activation via cAMP/PKA-mediated stimulation of AREG and EREG expression [7], amplifying ERK1/2 signaling and its effects on TZP dynamics. AKT, AKT serine/threonine kinase; AREG, amphiregulin; ARP, actin-related protein; CAM, calmodulin; cGMP, cyclic guanosine monophosphate; EGFR, epidermal growth factor receptor; EREG, epiregulin; ERK, extracellular signal-regulated kinase; FSHR, FSH receptor; IP3, inositol triphosphate; MLC, myosin light chain; NPPC, natriuretic peptide C; NPR2, natriuretic peptide receptor 2; PI3K, phosphoinositide-3 kinase; PKA, protein kinase A; PLC, protein kinase C; TZP, transzonal projections. Figure and legend text adapted from Buratini et al. (2022).

Curiously, while suppressing *AREG* and *EREG* mRNA expression, OSFs increased *EGFR* mRNA levels in bovine cumulus cells, suggesting that, although the oocyte is not in a hurry to resume meiosis and tries to enhance/prolong TZP-mediated communication with cumulus cells, it stimulates cumulus responsiveness to EGF-like factors for when the right time for the EGF-mediated trigger comes. We speculate that oleic acid may contribute to both aspects of this apparently paradoxical control, first by enhancing TZP-mediated communication via FSHR suppression and subsequently by stimulating EGF signaling at the right time. See Figure 3.

## In conclusion

Saturated FFA and unsaturated oleic acid have contrasting effects on steroidogenesis in the periovulatory follicle. Saturated FFAs, such as palmitic and stearic acid, appear to activate aromatase and increase E2 synthesis, while oleic acid induces a downregulation of E2 synthesis in granulosa cells, both in vivo and in vitro. Additionally, exposure to oleic acid leads to a decrease in FSHR expression, contrasting with saturated FFA, and may support communication between cumulus cells and the oocyte and prevent TZP retraction until the time is right. The *pERK* pathway activated by oleic acid in granulosa cells appears to promote the necessary switch from E2 dominance towards P4 dominance following the LH surge. The activation of the *pERK* pathway by oleic acid and its potential influence on the COC, particularly concerning its known connection to oocyte competence, possibly through a pathway triggered by the oocyte via FGF10/SCD/Oleic acid is an area for further investigation. The above data suggest a potential regulatory, interconnected system involving oleic acid that modulates the steroidogenic switch in the periovulatory follicle, supporting the well-orchestrated dialogue between the oocyte and cumulus cells during the final maturation of COCs, from the LH surge to ovulation.

## Data Availability

No research data was used.
